# Resident macrophages of the lung and liver: The guardians of our tissues

**DOI:** 10.3389/fimmu.2022.1029085

**Published:** 2022-11-30

**Authors:** Amelia Kulle, Ajitha Thanabalasuriar, Taylor S. Cohen, Marta Szydlowska

**Affiliations:** ^1^ Department of Pharmacology and Therapeutics, McGill University, Montreal, QC, Canada; ^2^ Late Stage Development, Vaccines and Immune Therapies (V&I), BioPharmaceuticals R&D, AstraZeneca, Gaithersburg, MD, United States; ^3^ Bacteriology and Vaccine Discovery, Research and Early Development, Vaccines and Immune Therapies (V&I), BioPharmaceuticals R&D, AstraZeneca, Gaithersburg, MD, United States

**Keywords:** macrophages, liver, lung, homeostasis, inflammation, Kupffer cells, alveolar macrophages, interstitial macrophages

## Abstract

Resident macrophages play a unique role in the maintenance of tissue function. As phagocytes, they are an essential first line defenders against pathogens and much of the initial characterization of these cells was focused on their interaction with viral and bacterial pathogens. However, these cells are increasingly recognized as contributing to more than just host defense. Through cytokine production, receptor engagement and gap junction communication resident macrophages tune tissue inflammatory tone, influence adaptive immune cell phenotype and regulate tissue structure and function. This review highlights resident macrophages in the liver and lung as they hold unique roles in the maintenance of the interface between the circulatory system and the external environment. As such, we detail the developmental origin of these cells, their contribution to host defense and the array of tools these cells use to regulate tissue homeostasis.

## Introduction

Tissue resident macrophages are long-lived innate immune cells that persist within various tissues in the body. These cells play many roles in health and disease and are unique when compared to more transient innate immune cells not just in function but also genetic profile. Herein we explore the origins and functions of tissue-resident macrophages, with a focus on those populations found in the lung and liver. Moreover, we define the role tissue resident macrophages play in maintaining tissue homeostasis and the ability of these cells to drive disease when improperly regulated.

Macrophages are a type of innate immune cells known for their ability to phagocytose debris and pathogens and present antigens to direct adaptive immunity. The discovery of macrophages is credited to Ilya Metchnikoff, a French-Russian zoologist who identified the cell population while studying phagocytosis (1). Shortly thereafter, macrophages were designated as tissue phagocytes within the reticuloendothelial system which proposed that endothelial cells and reticulocytes, otherwise known as phagocytes, shared a common origin (2). However, with the identification of morphological and functional differences between phagocytes and endothelial cells, this theory did not accurately depict cell lineage. Until recently, the prevailing belief was that all macrophages are terminally differentiated cells derived from circulating macrophages and monocytes (3). This belief was first proposed by Ralph van Furth in the 1960s and led to the establishment of the mononuclear phagocyte system (4). Although contradictory evidence has been published for many years, recent technological advances have allowed the definitive rejection of the mononuclear phagocyte system and the recognition of tissue-resident macrophages as unique populations independent of monocyte differentiation (5–7).

Herein we review the current understanding of resident macrophage populations in two major organs: the lung which is constantly exposed to the external environment and the liver which acts to filter the blood as it circulates through the body. In addition we cover the origin and maintenance of these cell populations, their role in maintaining tissue homeostasis and defense against invading pathogens. Finally, we discuss the implications of resident macrophage dysfunction in various diseases.

## Origins

Evidence of long-term persistence and proliferation in tissues led researchers to investigate the origins of tissue-resident macrophages. Several studies provided this evidence through the analysis of macrophages in organ transplant patients which demonstrated long-term maintenance of the donor macrophage population years after surgery (8–10). From there, evidence of macrophage populations in the yolk sac before the establishment of monocyte precursors suggested an embryonic origin for some macrophages (11). Indeed, further studies elucidated that tissue-resident macrophage populations in most organs of both mice and humans are established during embryogenesis and maintained through self-renewal (12). Classically, mature fully differentiated cells lose the capacity to proliferate as differentiation coincides with cell cycle withdrawal. Therefore, tissue-resident macrophages are unique in their ability to re-enter the cell cycle as fully differentiated cells (13). It must be noted that most findings on tissue-resident macrophage origins were established in mice as the study of tissue-resident macrophage ontogeny in human organs is limited (14).

Most mammalian organs have populations of tissue-resident macrophages. Some of the most well-known include Langerhans cells in the skin, alveolar macrophages in the lung, Kupffer cells in the liver, microglia in the brain, and red pulp macrophages in the spleen. These cells take on a number of different functions, that at first were defined by M1 or M2 polarization (15, 16). M1-macrophages are defined by a pro-inflammatory polarization as they secrete higher levels of pro-inflammatory factors and expression of the surface marker CD86. The second population, M2-macrophages, were thought to be immune-suppressive, support tissue repair, and characterized by the surface marker CD206 (17). More recently, it became clear that this paradigm does not mirror the high level of plasticity in the polarization of these cells, that often show a spectrum of features typical of M1 or M2 cells. For example, four different subtypes of M2 polarization were recognized: M2a-M2d, together with M4, Mox and M(Hb) polarization types based on the activating factors and function of each of mentioned populations (18). Resident cells respond to several different stimuli by modifying both function and surface marker expression, and as such are referred to as plastic.

Based on mouse studies, tissue resident macrophages, are generated in three waves. The first wave referred to as the primitive wave begins on embryonic day 6.5 (E6.5) in the yolk sac (19). This wave establishes a population of macrophage-exclusive progenitors which then differentiate into erythroid and myeloid progenitors (EMPs) between E8.5 and E10.5 during the pro-definitive wave (20). An intermediate pre-macrophage population (p-Macs) are derived from EMPs without first becoming monocytes (21). The population of EMPs and p-Macs expand for several days in the yolk sac and migrate to the fetal liver by E14.5. Between E12.5 and E17.5, p-Macs in the fetal liver migrate and seed other tissues to establish life-long populations of tissue-resident macrophages. The third wave of hematopoiesis, the definitive wave, begins around E17.5 and involves the establishment of hematopoietic stem cells in the bone marrow. Although all tissue-resident macrophages originate from fetal liver p-Macs, bone marrow derived macrophages are found to replace the fetal population in the intestines, spleen, skin, and heart (22). However, it must be noted that these hematopoietic stem cell derived macrophage populations are maintained through self-renewal with minimal contribution from circulating monocytes following their colonization of the tissue (23).

**Figure 1:**
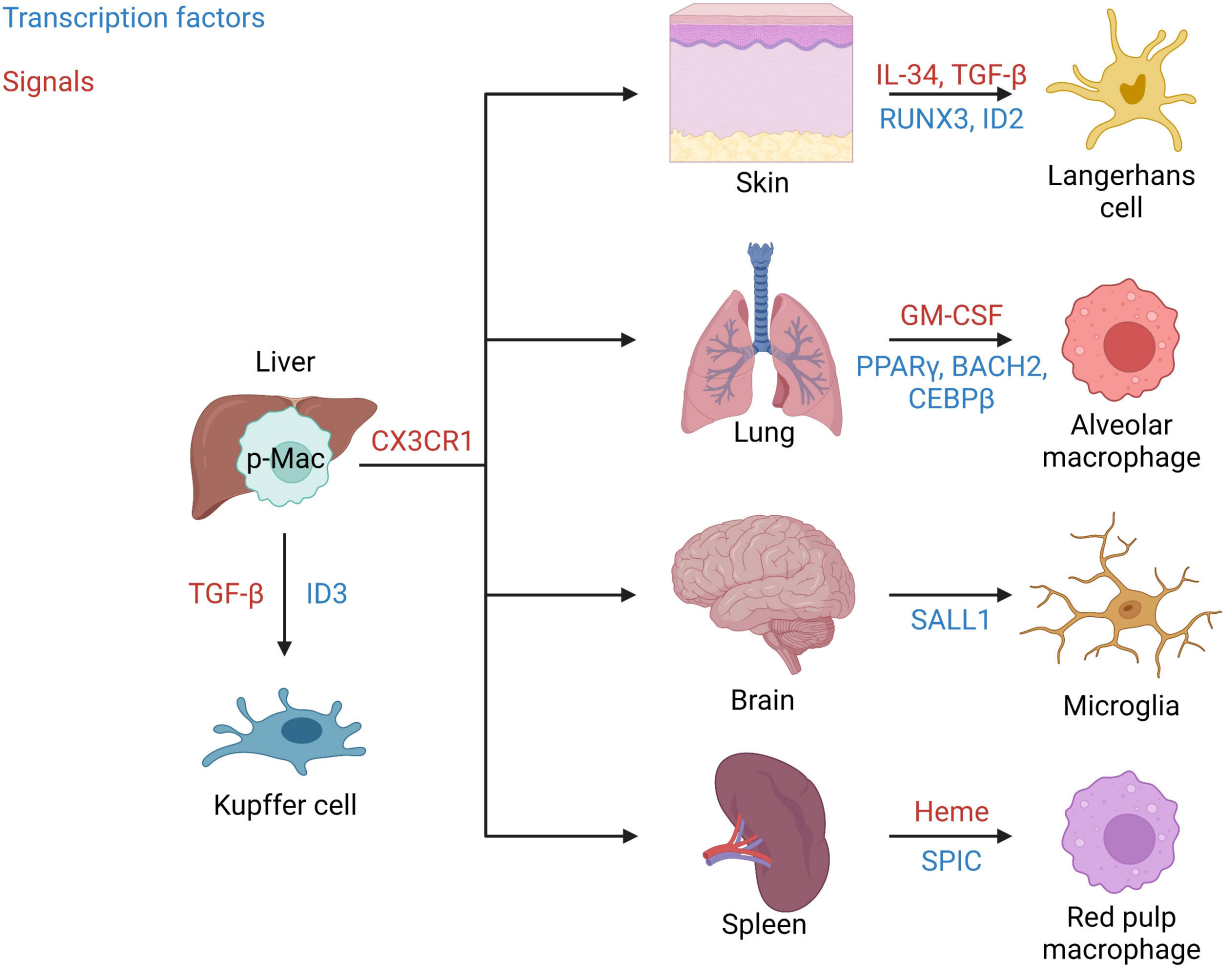
Tissue seeding. Signals and transcription factors involved in seeding of well-known tissue-resident macrophages from fetal liver p-Macs. Migration from the liver occurs in a CX3CR1 dependent manner. In the skin IL-34 and TGF-β induce the transcription factors RUNX3 and ID2 to generate Langerhans cells. In the lung GM-CSF induces the transcription factors PPARγ, BACH2, and CEBPβ to produce alveolar macrophages. In the brain/central nervous system, microglia are generated in response to the induction of the transcription factor SALL1. Red pulp macrophages in the spleen are produced in response to Heme and the transcription factor SPIC. Finally, in the liver Kupffer cells are generated in response to TGF-β and the transcription factor ID3.

Once seeded, tissue-resident macrophage mobility is restricted to their colonized tissue. Intriguingly, during the process of seeding embryonic tissues they are highly mobile. Therefore, researchers have wondered whether early macrophages are committed to a specific tissue before leaving the fetal liver, or if all tissues are seeded blindly and local cues guide tissue-specification? As mentioned, p-Macs begin to colonize embryonic tissues around E12.5. The migration of p-Macs is dependent on the expression of the chemokine receptor CX3CR1, which is not expressed by EMPs (24). In a study by Mass et al., single cell sequencing found that p-Macs lack tissue-specific signatures, thus suggesting that tissue-resident macrophages gain their specialized phenotype after colonization (21). Macrophage development is known to be regulated by the transcription factor PU.1 and is involved in tissue-resident macrophage specification by acting as a scaffold for histone modifiers that can induce chromatin remodeling (25–27). Once p-Macs seed a tissue, tissue-specific signals initiate the enrichment of transcriptional regulators to generate tissue-resident macrophages with specialized functions (**Figure 1**) (28). For example, the tissue-specific signals required for Langerhans cell development include IL-34 and TGF-β which induce the expression of the transcription factors RUNX3 and ID2 (29). In the lung, alveolar macrophages develop in response to granulocyte-macrophage colony-stimulating factor (GM-CSF) causing the upregulation of the transcription factor PPARγ. In addition, the transcription factors BACH2 and CEBPβ have been implicated in alveolar macrophage development (24, 30). Kupffer cell development in the liver has been found to be dependent on the transcription factor ID3 which is regulated by TGF-β (21). In the central nervous system, SALL1 is a microglia-specific transcription factor involved in their development (31). Finally, splenic red pulp macrophage development is induced by the presence of heme causing an upregulation in the transcription factor SPIC.

The remainder of this review will highlight the roles of tissue-resident macrophages in the liver and lung at steady-state and in disease. However, a comprehensive list of all tissue-resident macrophages, their origin, and functions is detailed in **Table 1**. The importance of the liver in our understanding of tissue-resident macrophages begins with its role as the reservoir of p-Macs to seed other tissues. In addition, during homeostasis it also maintains its own population of tissue-resident macrophages. In addition to the liver, the lung and brain are the only other tissues in which the population of tissue-resident macrophages is not replaced by hematopoietic stem cell derived macrophages following the initial colonization by fetal liver p-Macs. As such, these tissues have unique embryonic macrophage populations unlike the rest of the body. However, we will not be discussing tissue-resident macrophages in the brain as the blood-brain barrier isolates these macrophages from the external environment. In contrast the liver and lungs act as an interface between our circulatory systems and the external environment. Therefore, the tissue-resident macrophages in these organs play an essential role in host defense at steady-state as pathogens that enter circulation can cause systemic complications.

**Table 1:** Summary of tissue-resident macrophage subsets, origins, and functions.

Tissue	Subset	Origin	Functions	References
**Skin**	Langerhans Cells	Fetal	Immunosurveillance Present antigens to T cells Aid in hair follicle regeneration and tissue repair	**(** 32–34 **)**
	Dermal Macrophages	Adult	Immunosurveillance Aid in local nerve regeneration	**(** 35, 36 **)**
**Central Nervous System**	Microglia	Fetal	Immunosurveillance Neuronal development and synapse formation	**(** 37–39 **)**
**Lung**	Alveolar Macrophages	Fetal	Immunosurveillance Clearance of surfactant and inhaled pathogens	**(** 40–42 **)**
	Interstitial Macrophages	Adult	Immunoregulation	**(** 43, 44 **)**
**Spleen**	Red Pulp Macrophages	Fetal	Immunosurveillance Erythrocyte and iron recycling	**(** 45, 46 **)**
	Marginal Zone Macrophages	Requires further research	Immunosurveillance Clearance of pathogens in circulation and apoptotic cells	**(** 47–49 **)**
**Liver**	Kupffer Cells	Fetal	Immunosurveillance Iron, bilirubin, and cholesterol metabolism Clearance of gut-derived pathogens and damaged erythrocytes	**(** 50–54 **)**
	Capsular Macrophages	Adult	Immunosurveillance	**(** 55 **)**
**Bone**	Osteoclasts	Fetal/Adult	Resorption of bone matrix for bone remodeling	**(** 56 **)**
**Intestine**	Intestinal Macrophages	Fetal/Adult	Immunosurveillance Regulate smooth muscle contraction	**(** 57, 58 **)**
**Peritoneum**	Peritoneal Macrophages	Fetal	Immunosurveillance	**(** 59 **)**
**Heart**	Cardiac Macrophages	Fetal	Immunosurveillance Sustain myocardial conduction Stimulate angiogenesis	**(** 60–62 **)**
**Adipose**	Adipose Tissue Macrophages	Fetal	Control insulin sensitivity Sustain thermogenesis Regulate adipogenesis and angiogenesis	**(** 63–65 **)**

## Tissue-resident macrophages in the liver

**Figure 2:**
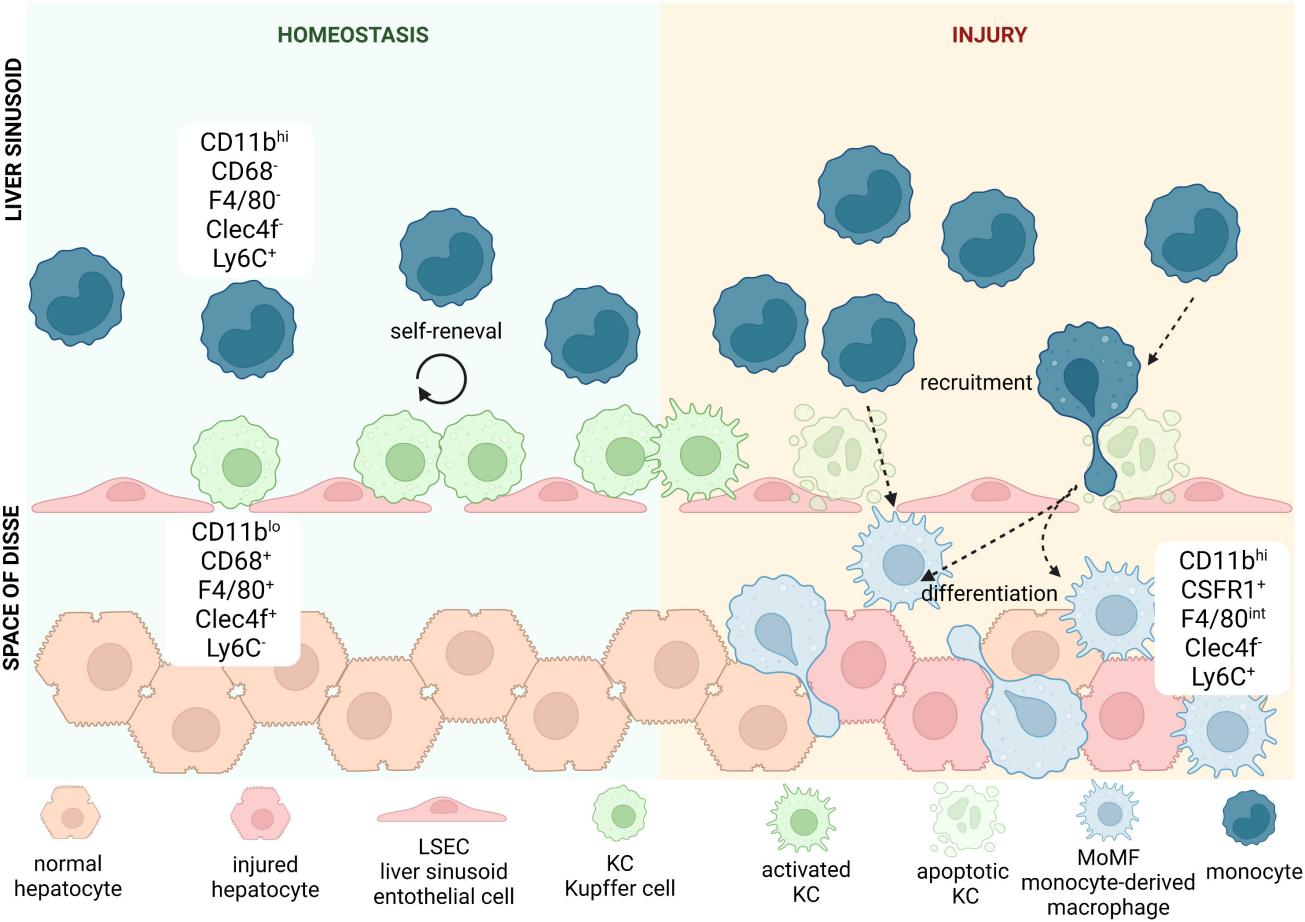
Heterogeneity of hepatic macrophages. In mice, liver-resident macrophages known as Kupffer cells (KCs) are classically defined by positive expression of CD11b, CD68, F4/80, Clec4f and negativity for Ly6C. They are located in liver sinusoids, where they adhere to liver sinusoid endothelial cells (LSECs). KCs thanks to their particular location remain in close contact with blood stream, which allows them to detect a variety of antigens. During **homeostasis** the pool of KCs is being replenished by cell renewal. During acute or chronic **liver injury** KCs get activated and secrete cytokines and chemokines that can recruit other immune cells from the circulating blood. Some of the secreted cytokines or chemokines thanks to fenestrae present between LSECs can reach liver parenchyma and directly affect hepatocytes and other immune cells located there. Haptic injury and/or chronic inflammation increases apoptotic rate of KCs and when self-renewal does not suffice to maintain their population, we can observe increased recruitment of monocytes, characterized by positivity for CD11b and Ly6C, with concomitant negativity to CD68, F4/80 and Clec4f. Once they enter parenchyma through endothelial fenestration, they differentiate into KC-like cells called monocyte-derived macrophages (MoMFs), that resemble the phenotype and function of KCs. These highly pro-inflammatory cells can be recognized by positivity for some markers typical for both KCs and monocytes: CD11b, F4/80 and Ly6C, while they remain negative for Clec4f. They also repopulate hepatic macrophages niche after increased death of KCs due to injury.

The fetal liver acts as a reservoir of tissue-resident macrophages, which then seed the organs during development, including the liver. It was long suspected that the population of macrophages in the liver consists of different subsets. So far, mostly thanks to preclinical studies performed on mice, researchers were able to characterize a few subpopulations based on origin and a high degree of phenotypic and functional specificity (**Figure 2**) (**Table 2**). In addition to the heterogeneity of the population, these cells are highly plastic and adapt to the dynamically changing liver microenvironment (66, 73–75). Classically, we would refer to two main subpopulations of macrophages in the liver, Kupffer Cells (KCs) and monocyte-derived macrophages (MoMFs). Kupffer cells (KCs) represent the major fraction of phagocytic cells in the liver at steady state (76, 77). The half-life of KCs in mice is estimated to be 12.4 days, while in humans transplanted donor-derived macrophages can be detected for as long as 1 year after surgery (78–80). Under physiological conditions, this population is replenished by self-renewal and does not depend on bone marrow-derived progenitors. The second subset, MoMFs, are not established embryonically, but serve to repopulate KCs during liver injury and/or chronic inflammation, where increased death of KCs, in most of cases apoptotic, can be observed (81–83). These newly recruited cells differentiate and acquire some of the phenotypic and functional features of KCs to replenish their population (67, 84). An additional population of hepatic macrophages reside in the capsule surrounding the whole organ referred to as liver capsular macrophages (LCMs), in mice expressing F4/80, CD11c and CX3XR1 and particularly enriched in CD207 in humans. This subset originates from adult circulating monocytes and are phenotypically distinct from other two populations, although further studies are needed to fully understand their role in liver homeostasis (55, 76).

**Table 2:** Basic markers of hepatic mononuclear phagocytes in mice and humans (55, 66–72).

	Mice							
Marker	KC1	KC2	MoMFs	Monocytes	LCMs	cDC1	cDC2	pDCs
CD11b	low	low	high	high	+	–	+	+
CD11c	–	–	–	–	low	+	+	–
CD68	+	+	–	–	–	–	–	–
F4/80	high	high	int	–	low	–	–	–
Clec4F	+	+	–	–	–	–	–	–
Tim4	+	+	–	–	–	–	–	–
MARCO	+	+	–	–	–	–	–	–
CD5L	+	+	–	–	–	–	–	–
VSIG4	+	+	–	–	–	–	–	–
Ly6C	–	–	high/int	high	–	–	+	–
CSFR1	+	+	+	+	+	–	–	–
CD206	low	high	–	–	–	–	–	–
ESAM	–	+	–	–	–	–	–	–
CD103	–	–	–	–	–	+/-	–	–
CD8α	–	–	–	–	–	+/-	–	–
CX3CR1	–	–	–	–	high	–	+	–
XCR1	–	–	–	–	–	+	–	–
MHC II	–	–	–	–	+	+	–	–
Siglec-H	–	–	–	–	–	–	–	+
CD317	–	–	–	–	–	–	–	+
	Human							
Marker	Pro-inflammatory KCs	Anti-inflammatory KCs	Intermediate Monocytes	Classical Monocytes	Non-classical Monocytes	pDCs	cDC1	cDC2
CD11b	+	+	+	+	+	+	–	+
CD11c	–	–	–	–	–	–	+	+
CD68	+	+	–	–	–	–	–	–
TIMD4	+	+	–	–	–	–	–	–
MARCO	–	+	–	–	–	–	–	–
CD5L	+	+	–	–	–	–	–	–
VSIG4	–	+	–	–	–	–	–	–
CD163	–	+	–	–	–	–	–	–
HMOX1	–	+	–	–	–	–	–	–
CD14	–	–	+	high	–	–	–	–
CD16	–	–	+	–	high	–	–	–
CCR2	–	–	+/-	–	–	–	–	–
HLA-DR	–	–	–	–	–	+	+	+
CD123	–	–	–	–	–	+	–	–
XCR1	–	–	–	–	–	–	+	–
CLEC9A	–	–	–	–	–	–	+	–
CD1c	–	–	–	–	–	–	–	+
SIRPα	–	–	–	–	–	–	–	+
CD172a	–	–	–	–	–	–	–	+
CD303	–	–	–	–	–	+	–	–
CD85g	–	–	–	–	–	+	–	–

KCs, Kupffer Cells; MoMFs, Monocyte-Derived Macrophages; LCM, Liver Capsular Macrophages; cDCs, Conventional Dendritic Cells; pDCs, Plasmocytoid Dendritic Cells; +, positive; -, negative; int, intermediate.

Liver macrophage populations can be distinguished from each other, and from infiltrating monocytes, by the differential expression of protein surface markers. Murine macrophages express CD11b and macrophage marker CD68, while a combination of other markers e.g., F4/80 and Clec4F (KC markers) or Ly6C (monocyte marker) help identify subpopulations (68). KCs can be identified by high expression of F4/80 and Clec4F, negative or low expression of Ly6C (CD11b^lo^CD68^+^F4/80^hi^Clec4F^+^Ly6C) and expression of scavenger receptors TLR4 and TLR9 (85). MoMFs are positive for CSF1R and Ly6C and express increasing levels of F4/80 as they differentiate into KC-like cells (CD11b^hi^CSF1R^+^ F4/80^int^Ly6C^hi/lo^). In parallel, levels of Ly6C decrease as they become KC-like (67, 69). LCMs residing in hepatic capsule share some of the surface antigens with both KCs (F4/80) and MoMFs (CSF1R), while also expressing the dendritic cell marker CD11c (55). Non-resident monocytes are also found in the liver and can be separated from resident cells based on negative expression of F4/80 or Clec4F and high expression of Ly6C (86).

In addition to these classical populations, recent data obtained using single-cell RNA sequencing (scRNA seq) and further corroborated by flow cytometry, proteomics and mechanistic studies led to a discovery of two distinct populations of KCs in the liver: KC1 (consisting of 85% of KCs) and KC2 (15%). While both share all markers typical for KCs, they can be distinguished by their differential expression of CD206 and ESAM with KC1 cells identified as CD206^lo^ and ESAM^-^ and KC2 as CD206^hi^ and ESAM^+^. The ratio between both populations in the hepatic tissue remains stable in the steady state, with similar localization in sinusoids and zonation. On the other hand, in-depth transcriptomic analysis revealed particular enrichment of KC2 cells in genes involved in carbohydrate and lipid metabolism, what suggested their role in liver metabolic disorders (87).

The definition and characteristics of different human hepatic macrophage populations are less clear due to the limited availability of appropriate samples, although recently published scRNAseq data shed more light into that topic and discovered multiple novel markers allowing identification of cell subsets (**Table 2**). The main subsets of monocytes and macrophages are identified by expression of CD68, MARCO, TIMD4, CCR2, CD14 and CD16 (67, 69–71). KCs are defined as CD68^+^TIMD4^+^ cells and some, MARCO^+^KCs, in addition express VSIG4, CD163 and HMOX1 that suggest an immune-tolerogenic or immunosuppressive role. scRNAseq revealed that apart from mentioned markers, these cells show as well enriched expression of e.g. *CD5L*, *VCAM1* or *KLF4* (66). Meanwhile CD68^+^MARCO^-^ macrophages produce IL-18 and have a transcriptomic profile associated with more pro-inflammatory polarization, as they are enriched in *LYZ, CSTA* or *CD74* genes (66, 67, 69, 88). CD14^hi^CD16^-^ are defined as classical monocytes, CD14^+^CD16^+^ as intermediate and CD14^-^CD16^hi^ as non-classical monocytes, with CD14^+^CD16^+/-^CCR2^+^ cells most likely corresponding to pro-inflammatory murine MoMFs (70). All of mentioned populations and markers for both murine and human liver macrophages, monocytes and dendritic cells are summarized in the **Table 2**.

## Macrophages in the liver during homeostasis and inflammation

Six main functions of hepatic macrophages have been observed. As immune cells they carry immune surveillance while maintaining immune tolerance (I) but also serve as a first line of anti-microbial defense (II) (89–91). Moreover, they perform the clearance of the cellular debris and metabolites (III), maintain the iron homeostasis through phagocytosis of red blood cells (RBCs) (IV) and regulate cholesterol metabolism (V) (50–54, 92, 93). Lastly, through the interaction with other cell types in the liver they are important players in hepatic tissue repair (VI) (94, 95). The liver is exposed to the nutrients, metabolites and bacteria absorbed in the digestive tract (96). Hepatic macrophages serve an essential role in monitoring the gut-liver axis for invading pathogens and toxins (90). KCs are the most abundant immune cell population in the liver, residing in hepatic sinusoids (97). KCs express classic pattern recognition receptors to identify pathogen-associated molecular patterns (PAMPs) and damage-associated molecular patterns (DAMPs), including LPS, bacterial wall parts, DNA and lipoproteins. Moreover, they express CRig receptor that are able to effectively catch bacteria from the blood stream (55, 98–105).

Although KCs play a crucial role in the activation of an inflammatory response, they are equally important players in immunotolerance. They are capable of not only suppressing activity of effector T cells, but they are able to activate regulatory T cells (106). Liver macrophages can recognize, digest, and dispose of apoptotic and necrotic bodies. Immediate reaction from phagocytes and removal of necrotic bodies is particularly important, as cell contents released during necrosis are potent activators of immune response, therefore could sustain inflammation and delay tissue regeneration (107). Apart from removing the cellular debris from the hepatic parenchyma, liver macrophages also play an important role in iron homeostasis through the removal of damaged or aged RBCs and vesicles containing hemoglobin (Hb). Their involvement in this process depends on their expression of scavenger receptors which recognize polyinosinic acid or phosphatidylserine. In addition, KCs take up Hb-containing vesicles which helps to prevent the undesired loss of iron that could lead to its deficiency, or excess release of iron to extracellular matrix that could cause iron-induced toxicity (52, 108). Lastly, KCs play a crucial role in the modulation of cholesterol metabolism. KCs ingest and transfer LDL-derived cholesterol to hepatocytes and maintain HDL and VLDL levels through the surface receptor cholesteryl ester transfer protein (CETP) (109, 110).

## Tissue-resident macrophages in the lung

**Figure 3:**
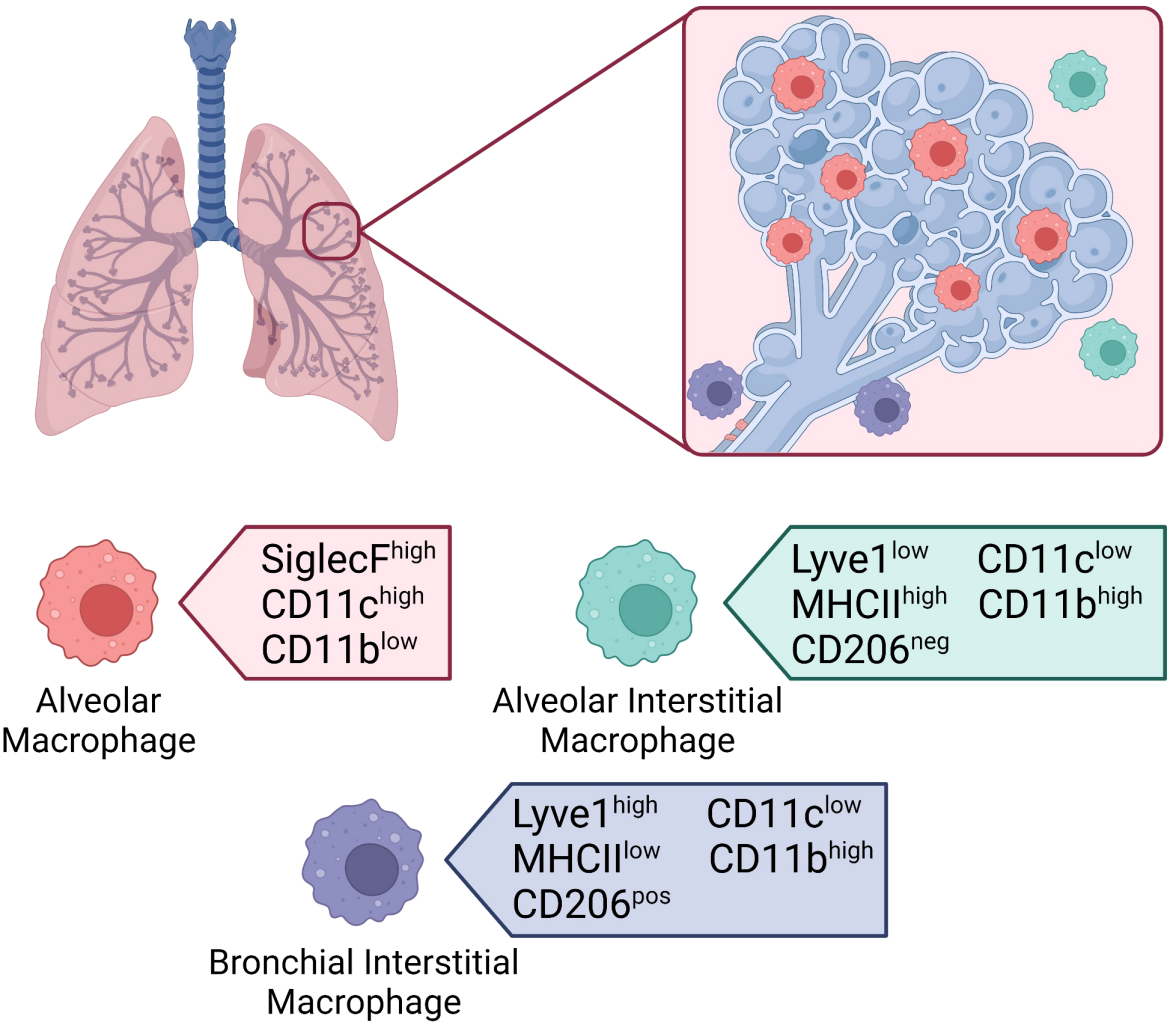
Macrophages in the lung. Location of tissue-resident macrophages in the lung and their distinguishing surface markers at steady-state. Alveolar macrophages located within the alveolar lumen express high levels of SiglecF and CD11c, and low levels of CD11b. Interstitial macrophages located in the alveolar interstitium express low levels of Lyve1, high levels of MHCII, and are CD206 negative. Interstitial macrophages located in the bronchial interstitium express high levels of Lyve1, low levels of MHCII, and are CD206 positive. Both interstitial macrophage subsets express low levels of CD11c and high levels of CD11b.

Tissue resident macrophages in the lung make the biggest journey in their life shortly after birth and are seeded from fetal liver macrophages within the first 3 days following birth (111, 112). The average person inhales more than 10,000 liters of air daily (113). As such, the lungs are constantly exposed to foreign particulates and pathogens from the external environment. Tissue-resident macrophages in the lung play a major role in filtering inhaled air and maintaining tissue homeostasis, to protect the host from airborne pathogens. Our lungs have two main populations of tissue-resident macrophages with specialized functions based on their anatomical compartment (**Figure 3**) (114). Alveolar macrophages (AMs) are found within the alveolus, while interstitial macrophages (IMs) are found in the surrounding tissue (115). Macrophages located within the upper airways have been proposed as a third population but are generally grouped with AMs (116). Our understanding of AMs is far more advanced than IMs as they are more accessible through bronchoalveolar lavage compared to tissue digestion required to isolate IMs. Recent technological advances have shed light on the importance of both subsets in maintaining the integrity of our lungs. We will explore the roles of AMs and IMs during homeostasis, infection, injury, repair, and disease.

## Lung resident macrophages in homeostasis and inflammation

### Alveolar macrophages

**Figure 4:**
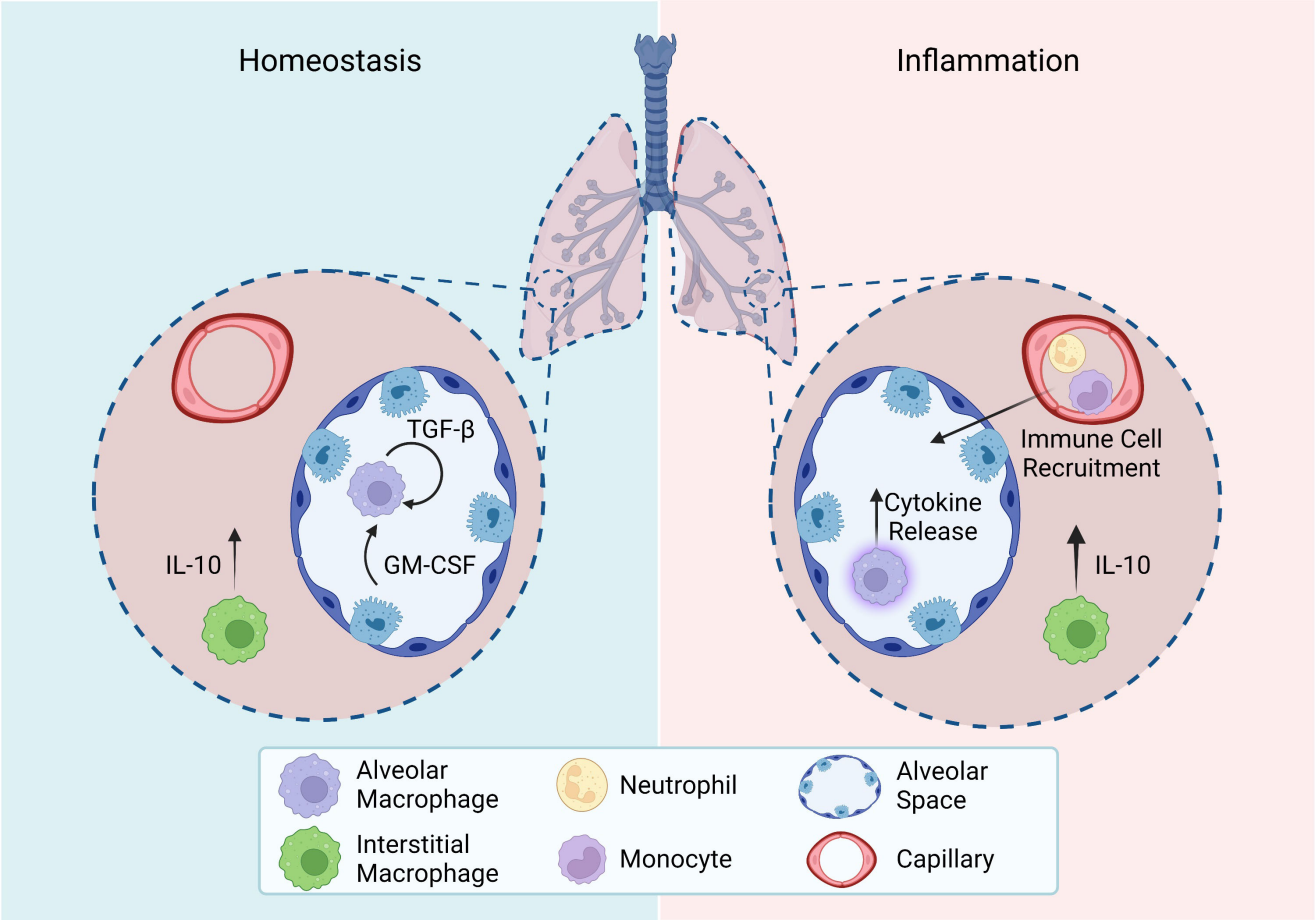
Homeostasis and inflammation in the lung. Roles of tissue-resident macrophage subsets in the lung during homeostasis and inflammation. Homeostasis: Alveolar macrophage maintenance relies on autocrine TGF-β and epithelial cell release of GM-CSF. Interstitial macrophages constitutively release IL-10. Inflammation: Activated alveolar macrophages release cytokines to recruit other immune cells and promote an inflammatory response. Interstitial macrophages upregulate their release of IL-10.*All figures were created with BioRender.com*.

AMs are the most abundant tissue-resident macrophages in the respiratory tract (8, 113). Shortly after birth, the AM population is established in response to the production of GM-CSF by alveolar epithelial cells (14). GM-CSF upregulates the transcription factor peroxisome proliferator-activated γ (PPARγ) in embryonic precursors triggering the terminal differentiation into AMs. The establishment of the AM population coincides with alveologenesis – the process by which the alveolar space is created. As such, AMs interact with alveolar epithelial cells to contribute to alveologenesis (115, 117). Maintenance of the population is dependent on GM-CSF from alveolar epithelial cells and transforming growth factor β (TGF-β) which is released by AMs themselves (**Figure 4**) (116). Although AMs are generally considered to be anti-inflammatory in nature, they are highly adaptable, and their phenotype is dependent on the surrounding microenvironment (115). Under homeostatic conditions, murine AMs can be identified by the high expression of Siglec F and the dendritic cell marker CD11c, and low expression of CD11b (**Figure 3**) (112). As different subsets of mononuclear phagocytes express similar markers, additional information on their defining markers and the differences between subsets in both mice and humans can be found in **Table 3**. Until recently, AMs were considered to be sessile like most other tissue-resident macrophages (122). However, *in vivo* intravital imaging has revealed that populations of AMs are motile and move between alveoli through pores of Kohn (123). The ability of AMs to move through the airways and between alveoli makes them one of the most unique tissue resident macrophages, who are classical static after being seeded.

**Table 3:** Defining markers of pulmonary mononuclear phagocyte subsets in mice and humans at steady state (118–121).

Mice							
Marker	AMs	Alveolar IMs	Bronchial IMs	Ly6C+ Monocyte Derived Cells	Ly6C- Monocyte Derived Cells	CD103+ Dendritic Cells	CD11b+ Dendritic Cells
CD11b	–	+	+	+	+	–	+
CD11c	+	+	+	–	int	+	+
CD24	–	–	–	–	–	+	+
CD64	+	+	+	int	int	–	–
CD103	–	–	–	–	–	+	–
CD206	+	–	+	–	–	–	+
Ly6C	–	–	–	+	–	–	–
Lyve-1	n/d	low	high	n/d	n/d	n/d	n/d
MARCO	+	–	–	n/d	n/d	n/d	n/d
MHCII	int	+	–	–	–	+	+
SiglecF	+	–	–	–	–	–	–
Human							
Marker	AMs	IMs	CD14+ Monos	CD1a+ Monocyte Derived Cells	CD1a- Monocyte Derived Cells	CD1c+ Dendritic Cells	CD141+ Dendritic Cells
CD1a	–	–	–	+	–	–	–
CD1c	–	–	–	+	+	+	–
CD11b	+	+	+	–	+	–	+
CD11c	+	+	+	+	+	+	+
CD14	–	+	+	–	+	–	–
CD16	+	+	+	–	–	–	–
CD36	+	+	+	–	–	–	–
CD64	+	+	+	–	–	–	–
CD141	+	–	–	–	–	–	+
CD163	+	+	+	–	–	–	–
CD169	+	–	n/d	–	–	–	–
CD206	+	+	+	+	+	–	–
HLA-DR	+	+	+	+	+	+	+
MARCO	+	+	+	–	+	–	–

AMs, Alveolar Macrophages; IMs, Interstitial Macrophages; Monos, Monocytes; +, positive; -, negative; int, intermediate; n/d, not determined.

Often referred to as housekeeping cells, AMs patrol the alveolar lumen phagocytosing debris to maintain homeostasis. AMs are also essential for the catabolism of surfactant proteins (113). By cleaning up the airways, AMs prevent unwarranted inflammatory responses to harmless particulates and maintain gas exchange. At steady state, AMs have several immunosuppressive functions. The release of TGF-β by AMs prevent their activation through an autocrine loop and convert naïve or activated T cells into regulatory T cells which are also immunosuppressive. In addition, AMs can release vesicles that suppress cytokine secretion from alveolar epithelial cells. Overall, AMs maintain tissue homeostasis by removing debris and preventing excess inflammation.

As mentioned, AMs are located within the airways making them the first immune cell to encounter inhaled pathogens. This localization enables AMs to control the intensity of the inflammatory response. Under states of infection or injury, AMs detect PAMPs and DAMPs through pattern recognition receptors including toll-like receptors (113). These signals are integrated with other stimuli to determine the strength of the resulting response. AMs then release either pro-inflammatory mediators such as IL-1β, IL-6, and tumor necrosis factor alpha (TNF-α), or anti-inflammatory mediators including IL-10 and TGF-β (**Figure 4**). Both pro- and anti-inflammatory AMs can exist at the same time and can be differentiated based on the expression of CXCL2 (124). Additionally, pro-inflammatory AMs are essential for recruiting other immune cells (**Figure 4**) (115). In the case of severe infection, injury, or inflammation tissue-resident AMs are depleted and can be supplemented by circulating monocytes. These recruited monocytes can gradually transition to resemble and act like AMs while the tissue-resident population is replenished by self-renewal. Although AMs play a role in the initiation of inflammation, they also contribute to inflammation resolution. The phagocytic properties of AMs are essential in clearing apoptotic cells to reduce tissue injury. Additionally, AMs release several repair mediators including resolvin, protectin, and amphiregulin which enhance healing.

### Interstitial macrophages

Previously, IMs were thought to be an intermediate subtype between recruited and tissue-resident AMs. However, recent advances such as single cell RNA sequencing have allowed researchers to gain a better understanding of their phenotype and function (125). IMs differ from AMs as the embryonically derived IMs present in the lungs before birth are gradually replaced by circulating monocytes and have a shorter life span (126). Two distinct populations of IMs have been identified in mice based on their location and expression of several surface markers at steady state, mainly hyaluronan receptor (Lyve1), major histocompatibility complex II (MHCII), and lung macrophage mannose receptor (CD206) (**Figure 3**). IMs residing in the bronchial interstitium express high levels of the receptor Lyve1, low levels of MHCII and are CD206 positive. IMs located in the alveolar interstitium express low levels of Lyve1, high levels of MHCII and are CD206 negative. Unlike AMs, both subsets of IMs express high levels of CD11b and low levels of CD11c. In addition, IMs express monocyte-specific markers, further proving that the population is replenished by circulating monocytes (43). Each subset has specific roles based on their location. IMs within the alveolar interstitium are involved in antigen presentation while those within the bronchial interstitium play a role in healing and repair (125). However, both subsets constitutively express the immunosuppressive cytokine IL-10, and thus contribute to immunoregulation. Although our understanding of IMs is not as advanced as AMs, some key functions and characteristics have been uncovered.

As with our knowledge on the function of IMs during steady state, our knowledge of their role during inflammation is not well understood. Upon encountering unmethylated CpG regions of bacterial DNA, the IM population expands and a subsequent increase in IL-10 is seen (**Figure 4**). These newly differentiated IMs express higher levels of the classic pro-inflammatory macrophage markers CD40, CD80, and CD86, compared to steady state IMs (127). IMs located within the bronchial interstitium can regulate the permeability of surrounding blood vessels to control the influx of immune cells into the lung. In addition, IMs have been found to possess greater antigen-presentation capabilities compared to AMs. Overall, in response to injury, IMs are anti-inflammatory in nature to maintain tissue homeostasis. One way in which this phenotype is mediated is through the release of Rspondin3 by endothelial cells, which promotes inflammation resolution (128). These functions are likely only the tip of the iceberg, and more work is needed to uncover further functions in inflammation.

### Tissue resident macrophages and diseases

Tissue resident macrophages make up a small portion of tissues cells but their effect on tissue homeostasis is mighty. If the function of tissue-resident macrophages is impaired, several disease states can develop (129). Diseases such as non-alcoholic steatohepatitis, alcoholic steatohepatitis, autoimmune hepatitis, and toxic liver injury worsen as a result of the overactivation and necroptosis of KCs (**Table 4**). However, KCs are also found to play protective roles in viral hepatitis and liver cancer such that their depletion results in a worse prognosis. In the lung, defective phagocytosis by AMs results in pulmonary alveolar proteinosis, an inflammatory condition caused improper catabolism of surfactant (14). In addition, dysfunctional phagocytosis is also seen in asthma, chronic obstructive pulmonary disease (COPD), and cystic fibrosis (**Table 5**). We will now explore how the tissue-resident macrophages in the liver and lungs are implicated in these disease states.

**Table 4:** Effects of disease on tissue-resident macrophages in the liver.

Liver disease	Exacerbating	Protective
**NASH**	- Increased activation and apoptosis of KCs - Increased recruitment of MoMFs - Increased production of pro-inflammatory (IL-1β, TNF-α, IL-6, IL-8 MCP-1) or pro-fibrogenic (TGF-β, PDGF-β) factors - Increased recruitment of other immune cells - Orchestrating immune response to PAMPs and DAMPs	- Removal of apoptotic, necrotic or senescent cells - Clearance of translocating from the gut bacteria
**ALD**	- Increased activation and apoptosis of KCs - Increased recruitment of MoMFs - Increased production of pro-inflammatory (IL-1β, TNF-α, IL-6, IL-8 MCP-1) or pro-fibrogenic (TGF-β, PDGF-β) factors - Increased recruitment of other immune cells - Orchestrating immune response to PAMPs and DAMPs	- Removal of apoptotic, necrotic or senescent cells - Clearance of translocating from the gut bacteria
**Viral hepatitis**	- Increased activation of KCs - Decreased expression and responsiveness of TLR2 and TLR3 in cells infected with HCV - Increased IL-10 and decreased IL-1β production in cells infected with HBV leading to immune tolerance	- Orchestrating recruitment and immune response from NK and NKT cells to limit infection
**AIH**	- Activation and necroptosis of KCs - Increased production of pro-inflammatory factors	- No data available
**Toxic injury**	- Increased activation and apoptosis of KCs - Increased recruitment of MoMFs - Increased production of pro-inflammatory factors (IL-1β, TNF-α, IL-6, IL-8 MCP-1) - Increased expression of immunosuppressive PD-L1	- Removal of apoptotic and necrotic cells
**Liver fibrosis**	- Increased activation and apoptosis of KCs - Increased recruitment of MoMFs - Increased production of pro-fibrogenic TGF-β and PDGF-β	- Production of MMP-9, -12 and -13 supporting fibrosis resolution
**Liver cancer**	- Increased production of pro-inflammatory factors creating pro-tumorigenic environment TAMs and MDSCs: - Increased production of IL-10 and expression of PD-L1 limiting cancer recognition by T cells - Increased production of TGF-β and PDGF-β promoting tumor proliferation and neoangiogenesis	- Clearance of hepatocytes that underwent oncogene-induced senescence at early stages

**Table 5:** Effects of disease on tissue-resident macrophages in the lung.

Asthma and Allergies	COPD	Cystic Fibrosis	Influenza
Impaired phagocytosis Increased release of IL-10 and IL-27 to dampen inflammatory response Promote production of regulatory T cells	Impaired phagocytosis Dysfunctional toll-like receptors prevent recognition of DAMPs and PAMPs Increased release of IL-8, TNF-a, and reactive oxygen species	Impaired phagocytosis Increased lysosomal pH Increased release of IL-10, TNF-a, IL-8, and IL-1B	Release of interferons, IL-6, and IL-12 Impaired antibacterial properties Premature apoptosis

### Non-alcoholic steatohepatitis (NASH)

NASH is a liver condition characterized by hepatic steatosis (intrahepatic lipid accumulation in >5% hepatocytes), accompanied by inflammation and fibrosis in the absence of excessive alcohol consumption (130). To date, multiple analyses of human, biopsy-derived liver samples have shown reduced numbers of KCs and increased MoMFs correlating with severity of inflammation, level of liver damage, and stage of fibrosis (131–134). NASH development and progression are associated with altered gut microbiome composition (dysbiosis) and increased intestinal permeability leading to increased levels of DAMPs and PAMPs reaching the liver. These molecules are one of the main activators of KCs through TLR4 and TLR9 signaling (135, 136). Moreover, lipid accumulation in liver parenchyma over time causes increased cell death and oxidative stress due to lipid peroxidation, that leads to the activation of an immune response in the liver (137, 138). In NASH, platelets and KCs are the first immune cells affected by lipid peroxidation. Activated KCs orchestrate recruitment of other immune cells to the liver and secrete pro-inflammatory cytokines and chemokines e.g., IL-1β, TNF-α, IL-6, IL-8, or MCP-1. Moreover, activated KCs also produce TGF-β and PDGF-β, both chemokines known for their pro-fibrogenic proprieties (139–143). In NASH, dietary lipids and cholesterol induce pro-inflammatory transcriptomic changes in KCs e.g., increased expression of Macrophage scavenger receptor 1 (MSR1) (144). Reversing some of mentioned changes could potentially provide some therapeutic benefit, as shown by the example of MSR1-blocking Ab, that attenuated the disease progression in experimental animal model (145). The crucial role of KCs in the development and progression of NASH was further demonstrated by cell depletion experiments (143, 146).

In addition to well-established changes in KCs during liver injury, recent discovery of their two new subpopulations: KC1 and KC2 shed a new light into particular roles of these cells in metabolic changes in the liver in NASH. KC2 (described in more detail above) show enrichment in genes involved in lipid and glucose regulation in steady state, which get further upregulated in metabolic disorders, e.g. hepatic steatosis. One of the most upregulated genes was CD36 known for its role in lipid uptake and oxidative stress. Indeed, experimental data showed that mice lacking KC2 macrophages in their hepatic tissue were protected from obesity, showed decreased oxidative stress and liver steatosis. This interesting finding requires further investigation, although the data collected so far suggests KC2 cells might become one of the important potential targets for the therapy of liver steatosis and/or NASH (87).

Constant activation of KCs during NASH results in the exhaustion and apoptotic death of these cells (81). In chronic inflammation self-renewal of the KC population is severely impaired, therefore MoMFs are recruited from the peripheral circulation to repopulate the tissue. MoMFs are able to acquire some of the features of KCs but show a highly pro-inflammatory phenotype that maintains liver inflammation and prevents its resolution (81, 143). Since the recruitment of inflammatory Ly6C^high^ MoMFs to liver parenchyma occurs in CCR2-dependent manner, its pharmacological inhibition by cenicriviroc could be a potential therapeutic strategy, since its use in mouse model of NASH significantly improved liver inflammation and fibrosis (88).

### Alcohol-associated liver disease

Alcohol-associated liver disease (ALD) is a spectrum of liver pathologies related to excessive alcohol consumption, ranging from simple steatosis, through alcoholic hepatitis (AH) or alcoholic steatohepatitis (ASH), to alcohol-associated cirrhosis (AC) potentially resulting in development of HCC. In addition, we can distinguish a more acute AH showing characteristics of acute-on-chronic liver failure (147). ASH is characterized by the same pathophysiological features as in NASH driven by excessive alcohol consumption (148). As observed in NASH, AH or ASH are both characterized by increased numbers and activation of macrophages in both human liver biopsies and in animal models, which translates to higher levels of produced pro-inflammatory cytokines and pro-fibrogenic factors (149, 150). Similarly, activation of liver macrophages in ASH results from steatosis-related lipotoxicity, increased gut permeability, and endotoxemia (151–153). In both conditions, KC activation by LPS is induced through TLR4, although it was shown that it occurs in a slightly different manner. In NASH, MyD88 is crucial for KC activation, while in ASH this occurs in MyD88-independent manner that involves type I interferon signaling through the IRF3-dependent pathway (154). The crucial role of liver macrophages in the pathogenesis of ASH was ultimately confirmed by results of KC depletion by Clodronate, which reduced liver damage in chronic-binge ethanol-feeding mouse model of ASH (155).

### Viral hepatitis

Hepatitis B and hepatitis C are chronic liver infections caused by the hepatitis B virus (HBV) and hepatitis C virus (HCV), respectively (156). Despite vaccine preventing HBV infection and available treatment for hepatitis C, both diseases have high prevalence putting numerous patients in risk for development of fibrosis/cirrhosis and HCC (157). The chronicity of viral hepatic diseases depends on ability of immune system to clear the virus and its persistence (158, 159). Since both HBV and HCV pose high-risk for laboratory personnel and these viruses do not infect rodents, most of the data about hepatic viral infections come from studies performed on mice infected with lymphocytic choriomeningitis virus (LCMV) or *in vitro* experiments with primary hepatocytes (160). In the early stages of infection, KCs recognize viral particles through TLR2 and present viral antigens to T cells. Additional KC activation occurs in response to IFNγ which is secreted by activated CD8+ cytotoxic T cells (161). Simultaneously, activated KCs secrete pro-inflammatory factors IL-1β, IL-6, TNF-α and CXCL8 that recruits NK and NKT cells to help limit the infection (162). The role of KCs in control of infection was further confirmed by increased viral dissemination and enhanced liver damage following KC depletion (161, 163).

Surprisingly, some data suggest KCs also play an important role in immune tolerance against the virus. It was shown that primary monocytes differentiated to M1- or M2-like phenotypes and treated *ex vivo* with HBV decreased production of the pro-inflammatory cytokine IL-1β and increased release of anti-inflammatory IL-10 (164). The decreased production of IL-1β resulted from inhibition of NLRP3 inflammasome activation by viral antigen HBeAg (165). Apart from limited cytokine production, monocytes and macrophages from patients with chronic hepatitis C have altered TLR responsiveness corresponding with reduced expression of TLR2 and TLR3 (163, 166). Altogether these data suggest that role of liver macrophages in viral hepatitis is complex and more studies are needed to better understand their role in these pathologies.

### Auto-immune hepatitis

Auto-immune hepatitis (AIH) is characterized by liver necro-inflammation with lymphoplasmacytic infiltrates in hepatic tissue and a presence of circulating autoantibodies. Immune cell driven inflammation is a central mechanism in the development and progression of AIH, and targeting this inflammation with immunosuppressive therapies is an effective treatment. Among the few reports discussing the role of KCs in AIH, most focused on the finding of hyaline droplets in the cytoplasm of these cells (167, 168). In adults this histological feature was shared between samples with AIH and primary biliary cholangitis (PBC). In pediatric patients it was a key indicator of diagnosis of AIH versus other pediatric liver diseases, although the exact function of these cells is unknown (167–169). One of the recent studies focusing on the role of KCs in AIH suggest that AIH is associated with dysbiosis and leaky gut, resulting in activation of necroptosis signaling through receptor interacting protein kinase 3 (RIPk3) in hepatic macrophages. Induction of necroptosis in KCs leads to the release of pro-inflammatory cytokines and chemokines resulting in the aggravation of inflammation (170). These first reports suggest liver macrophages might have an important role in pathogenesis of AIH, although further studies are needed to shed some light on mechanism of its development.

### Toxic liver injury

Acute liver injury usually results from an overdose of hepatotoxic agents, e.g., acetaminophen (known as APAP) or carbon tetrachloride (CCl_4_), resulting in extensive and irreversible hepatocyte damage with a high-mortality risk (171, 172). Necrosis occurring in the liver parenchyma leads to high levels of oxidative stress, together with DAMPs released from dying hepatocytes, stimulate KC activation. One of the recent studies showed, using scRNAseq, that APAP-induced liver failure leads to activation of around 51% of KCs. Further transcriptome analysis revealed that activated KCs differed from quiescent ones by upregulation of genes involved in not only immune response, but also in chemotaxis, cell migration, as well as interferon response (173). The latter is not surprising, since it was observed in all major liver pathologies, that activated KCs secrete a variety of pro-inflammatory factors that recruit and activate other cell types leading to acute inflammation (174). One of the key factors involved in the process of KC activation in APAP-induced liver injury is macrophage-inducible C-type lectin (Mincle). Mincle recognizes spliceosome-associated protein 130 (SAP130) released by necrotic hepatocytes. KCs are the main source of Mincle, and Mincle KO mice subjected to APAP overdose were less prone to liver injury. These animals showed fewer necrotic lesions, lower levels of alanine aminotransferase and aspartate aminotransferase in their plasma, and decreased production of IL-1β (175).

Interestingly, recent evidence suggest that KCs can also exhibit an immune-suppressive role during APAP-induced liver injury. In acute injury KCs and intrahepatic T cells express high levels of PD-1 and PD-L1, which suppresses the anti-bacterial function of liver macrophages. These data suggest that check-point inhibitors could restore anti-bacterial function of KCs by targeting PD-1/PD-L1 signaling (176). Additionally, IL-10 secreted by KCs directly affected surrounding hepatocytes, which express higher levels of CXCR2 in response. Mice lacking KCs, treated with high doses of APAP and a CXCR2 inhibitor were more susceptible to liver damage and had impaired liver regeneration, implicating role of hepatic macrophages in organ recovery in CXCR2-dependent manner (177, 178). Acute liver injury and inflammation observed during hepatotoxicity results in the cell death of large numbers of hepatic macrophages, resulting in dramatically increased recruitment of MoMFs and monocytes from the circulation, like the liver repopulation process observed in NASH or ASH (179).

### Liver fibrosis

Liver fibrosis is a process of pathological scarring occurring within liver parenchyma, as a result of sustained activation of hepatic stellate cells (HSCs) due to chronic hepatic injury occurring e.g. in NASH, ALD or viral hepatitis (180). Activation of HSCs leads to excessive deposition of extracellular matrix (ECM) consisting mainly of collagen, which affects hepatic architecture and flow of oxygen and nutrients, that can further accelerate the injury causing progression of fibrosis towards cirrhosis (181, 182).

Although HSCs are responsible for ECM deposits, multiple other cell types are involved in fibrogenesis, including damaged hepatocytes and activated immune cells. Among them, activated during injury KCs play an important role through the production of pro-fibrogenic cytokines and chemokines, including PDGF-β and TGF-β. At the same time cytokines and chemokines released from injured hepatocytes, activated HSCs and immune cells (e.g. CCl2, CCl5), lead to increased recruitment of CCR2^+^Ly6C^hi^ monocytes that further differentiate towards Ly6C^hi^ MoMFs with highly pro-inflammatory and pro-fibrogenic proprieties (179, 183, 184). In turn, these cells can, through PDGF-β, TGF-β and CTGF, further stimulate ECM production by HSCs and promote their survival by IL-1 β and TNF-α (182, 185). On the other hand, after the peak of fibrogenesis process, MoMFs start to differentiate towards more restorative cells that are a source of matrix metalloproteinases MMP-9, -12 and -13 that were associated with fibrosis resolution (186–188). Unfortunately, the latter can be counteracted by tissue inhibitors of matrix metalloproteinases (TIMPs) produced by activated HSCs, preventing resolution of fibrosis during chronic pathologies (186, 189). Indeed, dual role of liver macrophages in liver fibrosis largely depends on the stage of fibrogenesis. In the mouse model of liver fibrosis induced by treatment with CCl_4_ depletion of hepatic macrophages during the progression of the disease reduced scarring. On the other hands depletion of the same cells during the phase of resolution limited ECM degradation and hepatic recovery, making liver macrophages a complicated target for therapy of liver fibrosis (190).

### Liver cancer

Hepatocellular carcinoma (HCC) and intrahepatic cholangiocarcinoma (iCC) are the two most frequent primary liver malignancies, with HCC consisting of 70% of the cases (191). Tumors develop due to sustained injury of hepatocytes or cholangiocytes leading to continuous cell death and compensatory proliferation, increasing the risk of genetic mutations. Some of these mutations lead to cell cycle arrest and apoptosis, while others initiate the process of hepatocarcinogenesis increasing the growth rate of these cells (192, 193). Chronic inflammation due to higher levels of cytokines, chemokines, and growth factors present in liver parenchyma create a microenvironment that promotes liver cancer onset and progression (194, 195). In the context of HCC, KCs play a protective role in the early phases of tumor initiation. Upon liver injury, senescent hepatocytes secrete CCL2, which in turn recruits CCR2^+^ macrophages. These cells remove hepatocytes that underwent oncogene-induced senescence from liver tissue and prevent initiation of carcinogenesis (76, 196, 197). Conversely, increased levels of CCL2 and recruitment of CCR2^+^ macrophages correlate with increased tumor burden and poor prognosis, suggesting that these cells might play pro-tumorigenic role. This hypothesis was confirmed by studies in which a CCR2 antagonist, RDC018, suppressed the development of liver cancer (198, 199).

Non-resident macrophages, such as tumor-associated macrophages (TAMs), induce immune tolerance against tumors by producing high levels of IL-10 and increasing the expression of PD-L1, thus limiting the immune response of T cells to cancer cells (200, 201). Another subset of macrophages with similar proprieties are called myeloid-derived suppressor cells (MDSCs) and an increase in both TAM and MDSC numbers was associated with increased tumor burden and higher metastasis rate in both preclinical models and human patients (202). Moreover, multiple factors released by TAMs, TNF-α, IL-1β, and IL-6 support tumorigenesis by maintaining a pro-inflammatory environment (203). Others, like TGF-β or PDGF-β promote fibrogenesis and act as growth factors promoting tumor proliferation or neoangiogenesis in fully formed tumors that increases the supply of nutrients and oxygen within the tumor (204, 205).

Considering the important role of liver macrophages in the maintenance of immune tolerance against tumors, targeting these cells to reverse their M2 phenotype or use of anti-PD-L1 antibodies might be a potential therapeutic strategy for the treatment of liver cancer. Preventing liver macrophages from inducing immune tolerance could activate T and NK cells and provoke anti-tumor immunity leading to inhibition of tumor growth. This approach, together with therapeutic strategies that are already in place, could increase the efficacy of treatment leading to the increased survival and a better quality of life for patients.

### Chronic obstructive pulmonary disease (COPD)

Patients with COPD have increased numbers of macrophages within their airways and interstitium. However, the function of these macrophages is highly impaired. To begin, AMs in COPD show impaired expression of toll-like receptors that are essential for detecting DAMPs and PAMPs (206). In addition, these AMs have dysfunctional phagocytic abilities which can lead to increased inflammation (207). Increased levels of IL-8, TNF-α, reactive oxygen species, and matrix metalloproteinase 12 are produced by AMs in COPD patients (129). These factors exacerbate inflammation and cause tissue damage. As for IMs, little is known about their role in COPD. However, murine studies suggest that they contribute to the release of the pro-inflammatory factors TNF-α and IL-6 (208). Current therapeutic options focus on reducing the symptoms of COPD in lieu of reversing disease progression. Therefore, researchers have looked at targeting lung macrophages to restore lung function. Shifting macrophage polarization towards an anti-inflammatory phenotype by reducing oxidative stress and supressing pro-inflammatory mediators release have been found to restore the phagocytic function of lung macrophages thus improving disease pathogenesis (209, 210).

### Asthma

Asthma and allergic reactions are attributed to the dysfunctional response of T helper 2 (Th2) cells, and tissue-resident lung macrophages play an important role in the regulation and maintenance of these T cells in the lung (211, 212). Th2 cells secrete the cytokines IL-4, IL-5, and IL-13 and stimulate a type II immune response. As the primary immune cell responsible for responding to allergens and pathogens in the airway, AM dysfunction can by linked to the development of T-cell mediated pathologies such as asthma. As such, pathways more commonly linked to macrophage function, such as the inflammasome, have been demonstrated to influence Th2 and regulatory T-cell development (213). In a mouse model of allergic asthma, loss of NLRP6 prevented development of Th2 mediated lung inflammation (214). In response to inflammation AMs release IL-27, an essential regulator of airway hyperresponsiveness known to be reduced in individuals with asthma (114, 215–217). When AMs are depleted, the lack of IL-27 production delays inflammation resolution exacerbating allergic inflammation (218). The intrinsic expression of TGF-β by AMs and IL-10 by IMs aid in controlling responses to inhaled allergens through influence on regulatory T-cells (219). Resident macrophages also play a central role in the clearance of dead and dying cells, which if left in the lung can be a driver of inflammation. In murine models, the efferocytosis of apoptotic cells by AMs prevented development of asthma (220, 221). Defective efferocytosis in AMs is in part due to altered expression of Axl receptor kinase (222). Blocking this pathway has been shown to prevent pathology during viral asthma exacerbation, suggesting that targeting macrophage function could be a therapeutic opportunity for the treatment or prevention of asthma (223).

### Cystic fibrosis

Cystic fibrosis (CF) is a genetic disease caused by mutations in the cystic fibrosis transmembrane conductance regulator (CFTR), which results in exaggerated airway inflammation, airway edema and impaired host defense (224). Due to the primary function of CFTR as an epithelial ion pump, the role of macrophages in CF is somewhat understudied. In patients, macrophage derived factors including IL-10, IL-8, TNF-α, and IL-1β were increased (225, 226). In addition, macrophages in these patients show impaired phagocytosis and bactericidal killing due to an increased lysosomal pH (227, 228). Murine studies have further demonstrated that loss of CFTR influences macrophage function. AMs and bone marrow-derived macrophages from mice lacking CFTR produced higher amounts of inflammatory cytokines ex vivo following simulation with LPS, in part due to altered trafficking of TLR4 in these cells (229, 230). Interestingly, defects in phagocytosis or phagolysosome maturation have not been confirmed, suggesting that while the inflammatory response of the AM is altered in CF, their ability to eliminate bacteria may not be impacted (231, 232).

### Influenza

Influenza is a highly prevalent viral infection affecting an estimated 1 billion people every year. As with bacterial infections, AMs play a critical role in host defense. In response to the detection of PAMPs by TLRs, AMs release cytokines including interferons, IL-6, and IL-12 (233). The importance of AMs in combatting influenza has been shown through depletion experiments resulting in uncontrolled viral infection (234, 235). However, influenza can also have deleterious effects on the AM population. Verma et al., found that influenza activates IFNγR signaling in AMs resulting in reduced antibacterial abilities (236). This deficient antibacterial activity leaves the host vulnerable to secondary infections. In addition, influenza infections induce premature apoptosis and depletion of AMs (237). Impaired AM function can cause surfactant accumulation leading to respiratory distress. Overall, AMs can play a critical role in both the defense and pathogenesis of influenza infections. As such, AMs could be therapeutic target to lessen disease severity. The primary way in which AMs have been targeted has been to downregulate the release of pro-inflammatory cytokines from dysregulated macrophages to prevent lung injury (238, 239).

## Conclusion and future perspectives

Tissue resident macrophages are a unique sub-arm of the innate immune system. These cells guard our organs from infection and play pivotal roles in tissue homeostasis. Resident macrophages seed the major organs, such as liver and lung, during embryonic development, and they stay with the organ throughout life. These cells direct organ development, regulate the phenotype of other immune cells and protect the tissue from microbial infection. With such an essential role in directing organ function and protecting from infection, it is understandable that a number of diseases are associated with macrophage dysfunction. Our understanding of tissue resident macrophages is still limited, particularly chronic respiratory disease. Due to the myriad of functions driven by macrophages, they are an attractive target for therapeutic intervention. Additional research is required to more deeply understand the molecular signaling regulating macrophage function and to safely develop therapies directed at this essential cell type.

## Author contributions

AK and MS wrote the manuscript, AT, TSC, and MS revised the manuscript. All authors contributed to the article and approved the submitted version.

## Funding

AK was supported by The Fighting Blindness Canada Funding.

## Conflict of interest

TSC and MS are employed by AstraZeneca. 

The remaining authors declare that the research was conducted in the absence of any commercial or financial relationships that could be construed as a potential conflict of interest.

## Publisher’s note

All claims expressed in this article are solely those of the authors and do not necessarily represent those of their affiliated organizations, or those of the publisher, the editors and the reviewers. Any product that may be evaluated in this article, or claim that may be made by its manufacturer, is not guaranteed or endorsed by the publisher.
